# Endometriosis Patients Have an Increased Risk of Experiencing Long-Covid Symptoms: Results from a Cross-Sectional Multicenter Study

**DOI:** 10.1089/whr.2024.0049

**Published:** 2024-09-13

**Authors:** Anna Cirkel, Hartmut Göbel, Carl Göbel, Ibrahim Alkatout, Ahmed Khalil, Sascha Baum, Norbert Brüggemann, Achim Rody, Christoph Cirkel

**Affiliations:** ^1^ Department of Neurology, University Hospital Schleswig Holstein, Luebeck, Germany.; ^2^ Kiel Migraine and Headache Centre, Kiel, Germany.; ^3^ Department of Gynecology and Obstetrics, University Hospital Schleswig Holstein, Campus Kiel, Luebeck, Germany.; ^4^ Department of Gynecology and Obstetrics, University Hospital Schleswig Holstein, Campus Luebeck, Luebeck, Germany.; ^5^ Department of Gynecology and Obstetrics, Clinic Saarbrücken, Saarbrücken, Germany.

**Keywords:** menstrual pain, endometriosis, headache, long Covid, post-COVID-19, Sars-CoV-2

## Abstract

**Background:**

Women are more at risk for developing long-term symptoms after a COVID-19 infection. Only limited data are available for patients with coexisting endometriosis and/or menstrual pain symptoms.

**Study Design:**

A total of 840 premenopausal women with menstrual pain and/or endometriosis were included in this observational cross-sectional study using an online survey platform.

**Results:**

A total of 840 women with menstrual pain (mean age 30.7 ± 6.9, 15–54 years) were studied. Of these, 714 (84.2%) had a COVID-19 infection, 123 did not (14.5%). A total of 312 subjects had acute COVID-19 (AC) with symptoms ≤4 weeks (43.7%), 132 (18.5%) developed postacute COVID-19 syndrome (PC), and 88 (12.3%) had “*long Covid*” (LC). There were no statistical differences regarding number of vaccination shots between the three groups AC, PC, and LC. A total of 582 patients with surgically confirmed endometriosis (SCE) showed a twofold increased risk of LC [odds ratio (OR): 2.12, 2.18–3.84] in comparison with AC subjects. In SCE the comorbidity anxiety disorder (OR: 2.08, 1.14–3.81) and depression (OR: 2.02, 1.15–3.56) further increased the risk of LC. LC subjects had a significantly higher disturbance level of menstrual pain (*p* = 0.002), were more restricted in job (*p* < 0.001), leisure (*p* = 0.002), and family activities (*p* < 0.001), and had a higher number of endometriosis surgeries (*p* = 0.003).

**Conclusion:**

Subjects with SCE had a twofold increased risk of LC (in comparison to subjects with nonconfirmed endometriosis menstrual pain). In patients with SCE concomitant diagnosis of depression or anxiety disorder further twice-fold increased risk of LC. Further studies are needed if it is possible to reduce LC risk by improving the treatment of those secondary diagnoses and whether the type of endometriosis treatment can reduce LC occurrence (holistic, coanalgetic, hormonal).

## Introduction 

Infections with severe acute respiratory syndrome coronavirus 2 (SARS-CoV-2) caused coronavirus disease 2019 (COVID-19) pandemic. The number of patients with a COVID-19 infection increases and with it its associated long-term effects of this multiorgan disease with a wide spectrum of symptoms, manifestations, and long-term consequences.^1–5^ Meanwhile, the pandemic has started several years ago, and it is therefore eventually possible to show first long-term results, which are of considerable importance. Long Covid remains to be a worrisome issue for many patients, and it is therefore of importance to study risk factors of this disease. Long-term effects of COVID-19 are characterized by persistent or emerging symptoms in individuals with a history of probable or confirmed SARS-CoV-2 infection.^6^ Terminology still differs in the literature; however, it is commonly defined by disease duration,^6^ including (1) acute COVID-19 (AC) infection lasting up to four weeks, (2) ongoing symptomatic COVID-19 [sometimes called postacute COVID-19 syndrome (PC)^7^ or long-Covid syndrome,^8^ including signs and symptoms of four weeks up to 12 weeks], and (3) post-COVID-19 syndrome (“long Covid,” LC) shows signs and symptoms developing during or after infection consistent with COVID-19 being present for more than 12 weeks not attributable to other diagnoses.^7,8^ Other authors differentiate only between two groups instead of three: (1) acute COVID-19 (AC) infection (disease duration up to 4 weeks) and (2) postacute COVID-19 syndrome.^9^ In other works, long-COVID-19 syndrome—in contrast to the definition from the National Institute for Health and Care Excellence—is further defined as the continuation or development of new symptoms 12 weeks after the initial COVID-19 infection, with these symptoms lasting for at least two months with no other explanation.^7^

Symptoms of LC negatively impact the quality of life and commonly include multiple systems, including general symptoms (fatigue, fever), gynecological symptoms (pelvic pain, changes in menstrual cycles), neurological symptoms [headache, neurocognitive disturbances (often described as “brain fog”), anxiety, depression, sleep disturbances], respiratory and heart symptoms (dyspnea, cough, persistent oxygen requirement, chest pain, palpitations, thromboembolism), digestive and kidney symptoms (chronic kidney disease, diarrhea, abdominal pain), as well as other symptoms (hair loss, arthralgia, muscular weakness, rash).^9,10^ Postacute infection syndromes (PAIS) are not only known after COVID-19 infection, but are known after a vast variety of viral and nonviral infections.^9^ Although PAIS are known for a long time, little attention has been paid to them.^11^ Contributors to LC may include cellular damage, extensive immune response, including inflammatory cytokine production, and COVID-19 induced pro-coagulant state.^9^

Even though men show twice the risk of AC severity and mortality,^12^ women present with approximately 50% higher risk of developing LC.^13–16^ Specific comorbidities prior to AC infection are known risk factors for developing LC,^14^ among them migraine, endometriosis, anxiety, depression, and fibromyalgia.^14^ Women with endometriosis show a strong association with associated headache diseases or migraine [odds ratio (OR): 1.50, 95% confidence interval (95% CI): 1.29–1.74^17^]. Women with a history of endometriosis had a 22% higher risk of developing LC^14^ or a 1.22-fold increased risk compared with women without endometriosis.^16^ In addition, women with endometriosis who developed LC typically reported one additional Covid symptom in comparison with women without endometriosis.^16^

We therefore conducted this study to further evaluate the risks for developing LC in women with dysmenorrhea and surgically confirmed endometriosis (SCE) and their concomitant diseases to answer the following questions:

1. Do concomitant diseases have an effect on the likelihood of developing LC?
2. Does surgical confirmation of endometriosis have an impact on LC probability?
3. Does the number of COVID-19 vaccine shots differ in LC patients in comparison with patients without long-term effects after COVID-19 infection?
4. Do LC patients differ in clinical pain characteristics?

## Methods

This study was approved by the Ethics Committee (2023-287), and all subjects completed an informed consent statement before enrollment.

**FIG. 1.**
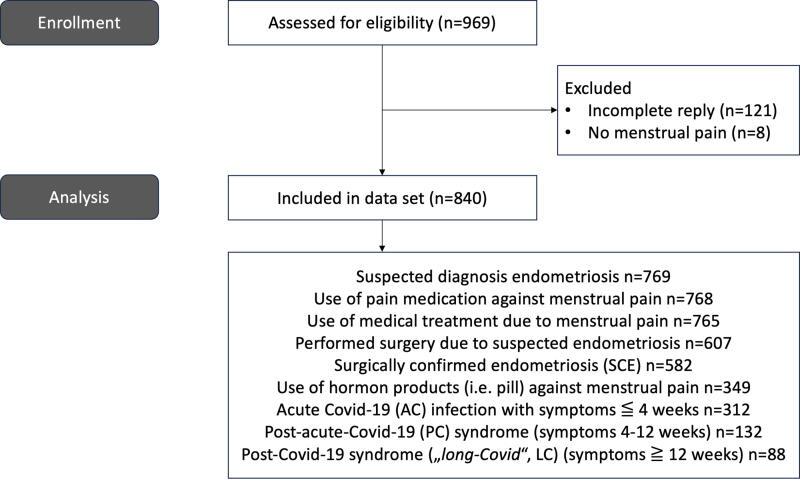
Flowchart of participant enrollment and analysis.

We conducted a cross-sectional multicenter study using an online survey platform (www.umfrageonline.com). Recruitment took place from May to November 2023. Patients from two German endometriosis centers were invited by email to participate in this study. The German Endometriosis Association posted an online invitation for participation on their homepage and social media. Inclusion criteria were as follows: women in between menarche and menopause who were in full command of the German language and could therefore understand the German survey. The study population consisted of women with menstrual pain; women who did not fit into this cohort were excluded from the study (see flowchart of enrollment and analysis after applying inclusion and exclusion criteria in Fig. 1).

An online survey was designed to assess our study aims. Before starting the survey, subjects were shown an informative text about our study aims, an average expected completion time, and were informed that the study was voluntary and anonymous. They were obliged to give informed consent for using and storing the anonymized data for research purposes. The online survey included the following sections: (1) Menstrual pain (*i.e.,* pain intensity, medication intake, burdens of pain), (2) suspected diagnosis endometriosis (*i.e.,* was the diagnosis suspected, was surgery performed), (3) sociodemographic and clinical data [*i.e.,* age, weight, height, further diagnoses (concomitant diseases included: migraine with and without aura, tension-type headache, depression, anxiety disorder, fibromyalgia, and chronic fatigue syndrome), education level], and (4) data regarding COVID-19 (*i.e.,* symptom duration, vaccination status).

Data analysis was performed with the Statistical Package for Social Sciences (IBM SPSS Statistics for MAC, Version 22.0. Armonk, NY: IBM Corp), and statistical significance was set at *p* < 0.05 (two-sided). Comparisons between groups were performed using one-way analysis of variance (one-way ANOVA) to compare between subjects who had (1) AC (symptoms of COVID-19 lasting <4 weeks), (2) PC (symptoms of COVID-19 lasting 4 until under 12 weeks), and (3) LC [symptoms of COVID-19 lasting >12 weeks (“long Covid”)]. None of the AC patients developed long-term effects after Covid infection. The crude OR at 95% CI and alpha level of significance set at 0.05 were used as measures of relative risk for developing LC (in comparison with AC) with different concomitant diseases. Results of the descriptive analysis were expressed as mean ± standard deviation (SD), absolute numbers for number of cases are also stated, and counting data are presented as the percentage of the total unless otherwise specified. Shapiro–Wilk test was performed to ascertain whether variables were normally distributed.

## Results

### Subjects

Among the 969 subjects who answered the questionnaire, a total of 840 subjects were included [129 (13.3%) excluded, see Figure 1] in the analysis after applying inclusion and exclusion criteria (all women, mean age 30.7 ± 6.9 years, range 15–54 years). In our study sample, 714 subjects reported to have had a COVID-19 infection (84.2%), and 123 reported to never have had it (14.5%) in the past. Criteria for PC were met by 132 subjects (18.5%), and for LC by 88 subjects (12.3%). Further characteristics of study participants are shown in Table 1. SCE was present in 582 subjects (69.3%), see Table 2.

**Table 1.** Variables Assessed in All Patients with Dysmenorrhea, Including Sociodemographic Data, Educational Status, Number of Vaccines Administered, Characteristics of Menstrual Pain, and Number of Received Surgeries for Suspected Endometriosis. Sample Size, Mean, and Statistical Test (ANOVA) Stated

Variable	Unit	*n* (%)	Acute COVID-19 (AC) infection (symptoms ≤4 weeks)		Postacute COVID-19 syndrome (PC) (symptoms 4–12 weeks)		Post-COVID-19 syndrome (“long Covid,” LC) (symptoms ≥12 weeks)		F	*p*
			*n* (%)	Mean ± SD	*n* (%)	Mean ± SD	*n* (%)	Mean ± SD		
All patients with dysmenorrhea		840 (100%)	312 (37.1%)		132 (15.7%)		88 (10.5%)			
Sociodemographic data										
Age	Years	840 (100%)	312 (37.1%)	29.65 ± 6.55	132 (15.7%)	31.31 ± 6.42	88 (10.5%)	31.24 ± 7.61	3.839	**0.022** ^f^
BMI	kg/m^2^	838 (99.7%)	310 (36.9%)	24.53 ± 5.07	131 (15.6%)	25.58 ± 5.46		25.34 ± 5.78	2.136	>0.05
Educational level	^a^	840 (100%)	312 (37.1%)	3.21 ± 0.87		3.13 ± 0.83		2.97 ± 0.81	2.997	>0.05
COVID-19 vaccination										
Number of COVID vaccines	^b^	840 (100%)	309 (36.8%)	2.81 ± 0.78	130 (15.5%)	2.79 ± 0.73	86 (10.2%)	2.71 ± 0.92	0.568	>0.05
Menstrual pain characteristics										
Intensity	VAS 0–10	840 (100%)	242 (28.8%)	7.26 ± 1.89	88 (10.5%)	7.52 ± 1.82	62 (7.4%)	7.74 ± 1.50	1.980	>0.05
Disturbance level	^c^		309 (36.8%)	4.15 ± 0.91	128 (15.2%)	4.29 ± 0.92	88 (10.5%)	4.51 ± 0.73	6.121	**0.002** ^f^
Intake of hormone products in past	No = 0, Yes = 1	Yes: 603 (71.8%)	Yes: 215 (25.6%)	0.69 ± 0.46	Yes: 97 (11.5%)	0.74 ± 0.44	Yes: 72 (8.6%)	0.82 ± 0.39	3.051	**0.048** ^f^
Present intake of hormone products	No = 0, Yes = 1	Yes: 349 (41.5%)	Yes: 131 (15.6%)	0.42 ± 0.49	Yes: 58 (6.9%)	0.44 ± 0.50	Yes: 35 (4.2%)	0.40 ± 0.49	0.154	>0.05
Restriction in job activities	^d^	840 (100%)	311 (37.0%)	2.41 ± 1.01	130	2.58 ± 0.98	88 (10.5%)	2.98 ± 0.01	11.620	**<0.001** ^f^
Restriction in leisure activities	^c^		312 (37.1%)	2.68 ± 0.95	131	2.89 ± 0.95		3.05 ± 0.84	6.179	**0.002** ^f^
Restriction in family activities	^c^			2.26 ± 1.03	130	2.48 ± 1.04		2.80 ± 0.94	10.003	**<0.001** ^f^
Number of surgeries										
Number of surgeries	^e^	767 (91.3%)	283 (33.7%)	1.10 ± 0.97	123	1.14 ± 0.90	82	1.52 ± 1.19	5.891	**0.003** ^f^
Migraine characteristics										
Frequency	n/month	149 (17.7%)	51 (6.1%)	3.92 ± 3.06	29 (3.5%)	4.07 ± 2.89	23 (2.7%)	6.39 ± 4.63	4.463	**0.014** ^f^
Intensity	VAS 0–10	321 (38.2%)	110 (13.1%)	7.10 ± 1.32	59 (7.0%)	7.31 ± 1.65	45 (5.4%)	7.56 ± 1.42	1.653	>0.05
Intake of medication	Days/month	299 (35.6%)	104 (12.4%)	2.69 ± 2.71	54 (6.4%)	2.74 ± 2.17	41 (4.9%)	4.80 ± 5.90	5.855	**0.003** ^f^
Restriction in job activities	^d^	320 (38.1%)	110 (13.1%)	2.08 ± 0.94	59 (7.0%)	2.29 ± 1.07	45 (5.4%)	2.47 ± 1.04	2.574	>0.05
Restriction in leisure activities	^c^			2.10 ± 0.92		2.46 ± 0.13		2.58 ± 0.99	5.091	**0.007** ^f^
Restriction in family activities	^c^			1.92 ± 0.93	58 (6.9%)	2.29 ± 0.99		2.51 ± 1.06	6.822	**0.001** ^f^
Combination of menstrual pain and migraine										
Restriction in job activities	^d^	185 (22.0%)	63 (7.5%)	2.76 ± 0.71	35 (4.2%)	3.09 ± 0.89	25 (3.0%)	3.28 ± 0.79	4.577	**0.012** ^f^
Restriction in leisure activities	^d^		64 (7.6%)	2.70 ± 0.79		3.06 ± 0.87		3.28 ± 0.74	5.339	**0.006** ^f^
Restriction in family activities	^d^	182 (21.7%)	61 (7.3%)	2.49 ± 0.74		3.00 ± 0.97		3.28 ± 0.74	9.763	**<0.001** ^f^

^a^ Secondary school (Hauptschule) = 1, intermediate school (Mittlere Reife) = 2, high school (Abitur/Fachabitur) = 3, university degree = 4.

^b^ 0 = never, 1 = once, 2 = twice, 3 = three times, 4 = four times, 5 = five times, 6 = more than five times.

^c^ No menstrual pain = 0, not disturbing = 1, marginally disturbing = 2, moderately disturbing = 3, severely disturbing = 4, very severely disturbing = 5.

^d^ Not at all = 0, slightly = 1, moderately = 2, severely = 3, very severely = 4.

^e^ None = 0, one = 1, two = 2, three = 3, four = 4, more than four = 5.

^f^ Significant results are highlighted in bold.

ANOVA, analysis of variance; BMI, body mass index; VAS, visual analogue scale.

**Table 2.** Variables Assessed in All Patients with Surgically Confirmed Endometriosis (SCE), Including Sociodemographic Data, Educational Status, Number of Vaccines Administered, Characteristics of Menstrual Pain, and Number of Received Surgeries for Suspected Endometriosis. Sample Size, Mean, and Statistical Test (ANOVA) Stated

Variable	Unit	*n* (%)	Acute COVID-19 (AC) infection (symptoms ≤4 weeks)		Postacute COVID-19 syndrome (PC) (symptoms 4–12 weeks)		Post-COVID-19 syndrome (“long Covid,” LC) (symptoms ≥12 weeks)		F	*p*
			*n* (%)	Mean ± SD	*n* (%)	Mean ± SD	*n* (%)	Mean ± SD		
All patients with surgically confirmed endometriosis (SCE)		582 (100%)	212 (36.4%)		91 (15.6%)		88 (10.5%)			
Sociodemographic data										
Age	Years	582 (100%)	212 (36.4%)	30.51 ± 6.64	91 (15.6%)	32.19 ± 5.72	72 (12.3%)	31.83 ± 7.93	2.417	>0.05
BMI	kg/m^2^	580 (99.7%)	210 (36.1%)	25.04 ± 5.37	90 (15.5%)	26.03 ± 5.63		25.72 ± 6.14	1.129	>0.05
Educational level	^a^	582 (100%)	212 (36.4%)	3.13 ± 0.92		3.08 ± 0.86		2.90 ± 0.81	1.722	>0.05
COVID-19 vaccination										
Number of COVID vaccines	^b^	582 (100%)	209 (35.9%)	2.88 ± 0.76	90 (15.5%)	2.79 ± 0.79	70 (12.0%)	2.71 ± 0.94	1.171	>0.05
Menstrual pain characteristics										
Intensity	VAS 0–10	582 (100%)	152 (26.1%)	7.16 ± 2.00	56 (9.6%)	7.57 ± 1.93	47 (8.1%)	7.66 ± 1.63	1.688	>0.05
Disturbance level	^c^		209 (35.9%)	4.12 ± 0.96	89 (15.3%)	4.28 ± 1.00	72 (12.4%)	4.47 ± 0.77	3.907	**0.021** ^f^
Intake of hormone products in past	No = 0, Yes = 1	Yes: 470 (80.8%)	Yes: 160 (27.5%)	0.76 ± 0.43	Yes: 71 (12.2%)	0.78 ± 0.42	Yes: 62 (10.7%)	0.86 ± 0.35	1.554	**0.048** ^f^
Present intake of hormone products	No = 0, Yes = 1	Yes: 279 (47.9%)	Yes: 131 (22.5%)	0.47 ± 050	Yes: 44 (7.6%)	0.48 ± 0.50	Yes: 31 (5.3%)	0.43 ± 0.50	0.250	>0.05
Restriction in job activities	^d^	582 (100%)	212 (36.4%)	2.42 ± 1.07	91 (15.6%)	2.69 ± 1.01	72 (12.4%)	2.90 ± 0.94	6.477	**0.002** ^f^
Restriction in leisure activities	^4^			2.66 ± 0.96		2.96 ± 0.98		2.96 ± 0.86	4.514	**0.012** ^f^
Restriction in family activities	^4^			2.25 ± 1.03		2.58 ± 1.07		2.72 ± 0.97	6.990	**0.001** ^f^
Number of surgeries										
Number of surgeries	^e^	582 (100%)	212 (36.4%)	1.44 ± 0.87	91 (15.6%)	1.52 ± 0.72	72 (12.3%)	1.71 ± 1.14	2.445	0.088
Migraine characteristics										
Frequency	*n*/month	110 (18.9%)	35 (6.0%)	3.74 ± 3.30	18 (3.1%)	4.61 ± 3.17	20 (3.4%)	6.25 ± 4.87	2.827	>0.05
Intensity	VAS 0–10	241 (41.4%)	77 (13.2%)	7.01 ± 1.24	41 (7.0%)	7.49 ± 1.55	38 (6.5%)	7.63 ± 1.44	3.151	**0.046** ^f^
Intake of medication	Days/month	224 (38.5%)	72 (12.4%)	2.32 ± 2.17	38 (6.5%)	2.55 ± 2.15	34 (5.8%)	5.06 ± 6.38	7.045	**0.001** ^f^
Restriction in job activities	^d^	240 (41.2%)	77 (13.2%)	2.04 ± 0.92	41 (7.0%)	2.37 ± 1.02	38 (6.5%)	2.42 ± 1.06	2.5555	>0.05
Restriction in leisure activities	^c^	241 (41.4%)		2.03 ± 0.92		2.46 ± 0.98		2.53 ± 0.98	4.758	**0.010** ^f^
Restriction in family activities	^c^	240 (41.2%)		1.90 ± 0.95	40 (6.9%)	2.28 ± 0.93		2.45 ± 1.06	4.670	**0.011** ^f^
Combination of menstrual pain and migraine										
Restriction in job activities	^d^	138 (23.7%)	42 (7.2%)	2.69 ± 0.64	25 (4.3%)	3.16 ± 0.85	21 (3.6%)	3.24 ± 0.83	5.007	**0.009** ^f^
Restriction in leisure activities	^d^		43 (7.2%)	2.65 ± 0.75		3.08 ± 0.86		3.24 ± 0.77	4.720	**0.011** ^f^
Restriction in family activities	^d^	136 (23.4%)	41 (7.0%)	2.44 ± 0.71		2.96 ± 0.94		3.24 ± 0.77	7.931	**<0.001** ^f^

^a^ Secondary school (Hauptschule) = 1, intermediate school (Mittlere Reife) = 2, high school (Abitur/Fachabitur) = 3, university degree = 4.

^b^ 0 = never, 1 = once, 2 = twice, 3 = three times, 4 = four times, 5 = five times, 6 = more than five times.

^c^ No menstrual pain = 0, not disturbing = 1, marginally disturbing = 2, moderately disturbing = 3, severely disturbing = 4, very severely disturbing = 5.

^d^ Not at all = 0, slightly = 1, moderately = 2, severely = 3, very severely = 4.

^e^ None = 0, one = 1, two = 2, three = 3, four = 4, more than four = 5.

^f^ Significant results are highlighted in bold.

### Comorbidities and odds ratio for experiencing LC symptoms

In the subgroup of dysmenorrhea, the surgical diagnosis of endometriosis leads to an increased OR of 2.12 (95% CI: 2.18–3.84) for experiencing LC symptoms. The comorbidity fibromyalgia has an OR of 3.19 (95% CI: 1.04–9.75). Depression and anxiety disorder increase the risk for LC with the OR of 1.90 (95% CI: 1.15–3.14) and 1.92 (95% CI: 1.12–3.30).

**FIG. 2.**
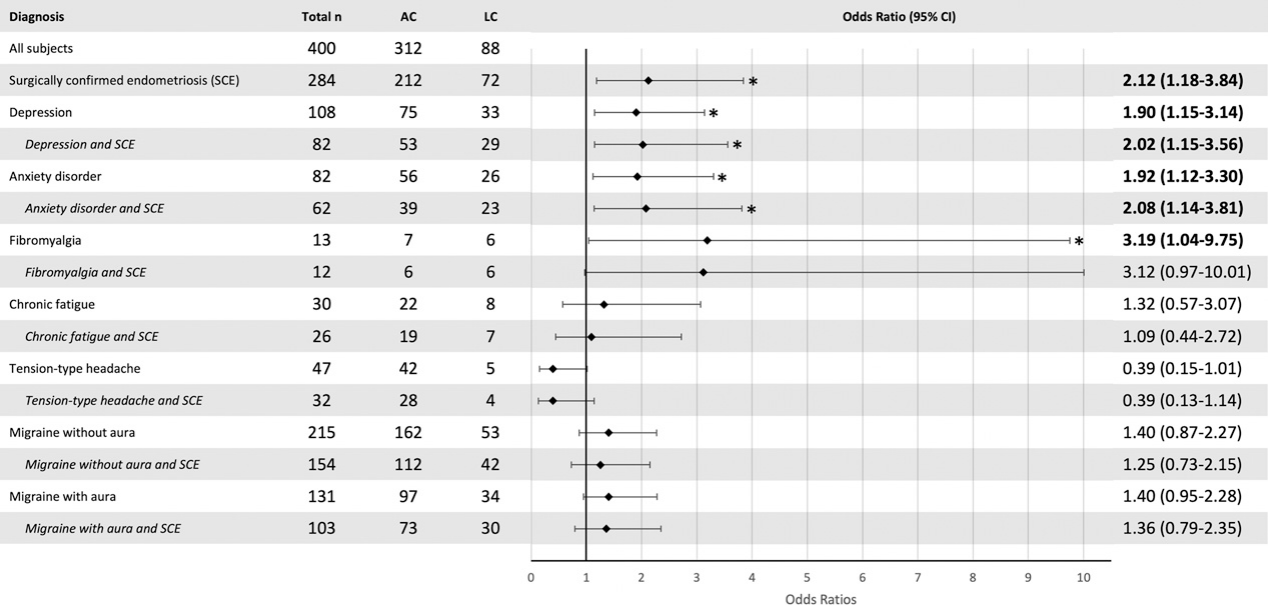
Analysis of relative risk for developing LC as a function of side diagnosis—in comparison with AC infection. Collective of SCE subjects is stated in italics. Significant results are highlighted in bold*. AC, acute COVID-19 infection with symptoms ≤4 weeks; CI, confidence interval; LC, post-COVID-19 syndrome or “long Covid” with symptoms ≥12 weeks; SCE, surgically confirmed endometriosis.

In patients with SCE depression [OR: 2.02 (95% CI: 1.15–3.56)] and anxiety disorder [OR: 2.08, (95% CI: 1.14–3.81)] further increase the risk of LC symptoms approximately by twofold. In SCE patients, the concomitant disease fibromyalgia did not increase the risk for LC. For all comorbidities see Figure 2.

### Sociodemographic and clinical characteristics

In the subgroup of dysmenorrhea, no significant differences can be seen in BMI and educational level when comparing AC, PC, and LC. However, significant differences are present for age, showing significantly older age in LC subjects (*F* = 3.839, *p* = 0.022) (see Table 1). Regarding COVID-19 vaccination, all three groups showed similar numbers of COVID-19 vaccine shots (minimum 2.71, maximum 2.81 shots; for further details, see Table 1). In the subgroup of SCE, no significant differences were seen for age, BMI, educational level, and number of COVID-19 vaccines (see Table 2).

### Menstrual pain conditions and COVID-19

In the subgroup of dysmenorrhea subjects, the disturbance level by menstrual pain was significantly higher in subjects experiencing LC (*F* = 6.121, *p* = 0.002), whereas there was no significant difference in menstrual pain intensity between groups. In addition, these subjects used endocrine dysmenorrhea treatments (*i.e.,* the pill) more often in the past (*F* = 3.051, *p* = 0.048) and are more restricted in job activities (*F* = 11.620, *p* < 0.001), leisure activities (*F* = 6.179, *p* = 0.002), and family activities (*F* = 10.003, *p* < 0.001) due to menstrual pain. The group of LC subjects has a history of more endometriosis surgeries (*F* = 5.891, *p* = 0.003) (see Table 1).

The results in the SCE subgroup show similar results (for further details, see Table 2): No significant difference in menstrual pain intensity, but significantly increased disturbance by menstrual pain (*F* = 3.907, *p* = 0.021), significantly more used endocrine dysmenorrhea treatments (*i.e.,* pill) in the past (*F* = 1.554, *p* = 0.048), and significantly more restrictions for job (*F* = 6.477, *p* = 0.002), leisure (*F* = 4.514, *p* = 0.012), and family activities (*F* = 6.990, *p* = 0.001). For number of surgeries, no significant differences could be seen in SCE subgroup.

### Migraine and COVID-19

Subjects with migraine and LC had a significantly higher migraine frequency (*F* = 4.463, *p* = 0.014) and used more pain medication against migraine (*F* = 5.855, *p* = 0.003), whereas migraine pain intensity did not significantly differ between AC, PC, and LC. Subjects with LC were significantly more restricted in leisure (*F* = 5.091, *p* = 0.007) and family activities (*F* = 6.822, *p* = 0.001) regarding their migraine (for further details, see Table 1).

In the case of additional SCE, LC subjects had a higher migraine intensity (*F* = 3.151, *p* = 0.046) and used more pain medication against migraine (*F* = 7.045, *p* = 0.001), whereas migraine frequency did not significantly differ between AC, PC, and LC. Subjects with LC were significantly more restricted in leisure (*F* = 4.785, *p* = 0.010) and family activities (*F* = 4.670, *p* = 0.011) regarding their migraine (see Table 2).

Subjects with dysmenorrhea and migraine symptoms at the same time that developed LC were significantly more restricted in job activities (*F* = 4.577, *p* = 0.012), leisure activities (*F* = 5.339, *p* = 0.006), and family activities (*F* = 9.763, *p* < 0.001) (see Table 1). If these subjects had SCE, they were also significantly more restricted in all three activities (see Table 2).

## Discussion

We evaluated the impact of long-term effects of COVID-19 in a cohort of women with known menstrual pain. To date, no study has explored potential influences of known menstrual pain conditions with duration of COVID-19 symptoms and relatively few studies examined the impact of LC on female reproductive health and associated illnesses.^18^

This is particularly interesting since premenopausal women have an increased risk for LC, which may suggest that sex hormones could play a role in LC development.^19–21^ In addition, endometriosis patients may have an increased risk of LC development.^14^

Microclots are typically present in LC,^22^ and it can be discussed that menstruation, female sex hormones, and/or hormonal treatment may increase hypercoagulation in LC. Patients with endometriosis also appear to be in a hypercoagulable state,^23^ possibly explaining the increased likelihood of endometriosis patients to obtain LC. Interestingly, estrogen and progesterone also show fibrinogen hypercoagulable properties.^24^ We conclude that more research is necessary to find out whether sex hormones influence hypercoagulation and microclotting in LC. However, since we have collected a cohort of SCE and endometriosis symptoms, we were also able to screen for subgroups, especially at risk of developing LC.

In our subgroup consisting of patients with menstrual pain, endometriosis symptoms, and SCE, we see that SCE, depression, anxiety disorder, and fibromyalgia increased the risk of experiencing LC. Our results further show that in patients with SCE, depression and anxiety disorder further increase the risk of LC. Patients with LC have significantly more endometriosis-related surgeries and have been treated more often with endocrine treatment. These patients also show a significantly higher disturbance level due to pain symptoms from dysmenorrhea. The disturbance of endometriosis symptoms, as shown by our results, might increase these patients’ need for either surgical or endocrine or both endometriosis treatments. Interestingly, there is no difference in the menstrual pain intensity, which might be explained by the fact that these patients at risk suffering from LC have a generally higher disturbance level of symptoms compared with subjects experiencing similar pain intensities. This is in line with our results regarding the impact on everyday activities. Subjects with LC also show significantly more restrictions in everyday activities in comparison with the other groups in regard to dysmenorrhea. If both dysmenorrhea and migraine symptoms were present at the same time, the subjects were also significantly more restricted in everyday activities. If migraine pain was at a different time than menstruation these patients also tended to be significantly more restricted in everyday activities. In addition, migraine as a comorbidity is a known risk factor for developing LC,^14^ which fits our results, showing that LC subjects required significantly more medication against migraine pain. It is possible that subjects with combined pain conditions (dysmenorrhea and migraine) are more sensitive to experiencing pain in general, explained by spatiotemporal reorganization of brain activity due to chronic pain conditions.^25^ It is well known that menstrual symptoms strongly affect the quality of life of females, including psychological and physical well-being, work life, and social life.^26,27^

Our results show that subjects with LC display significantly more restrictions in everyday activities due to menstrual pain and/or endometriosis in comparison with AC and PC and no differences were found in dysmenorrhea intensity, but they were found in pain experience. However, it remains unclear whether the differences in restriction in everyday activities originate from endometriosis or if this is caused by COVID-19. It is known that patients with LC have more adaptive coping styles.^28^

Future research should be performed to further study underlying mechanisms linking menstrual pain and/or concomitant diseases with LC development and whether it is possible to reduce the risk of LC by improving the treatment of those described conditions and whether the type of endometriosis treatment can reduce LC occurrence (holistic, coanalgetic, hormonal).

### Limitations

A limitation of our study is that COVID-19 infection, vaccination, and long-term symptoms were self-reported. Persistent physical symptoms after COVID-19 infection should not automatically be ascribed to the prior infection, instead, a detailed medical evaluation would be necessary. It therefore should be considered that symptoms were erroneously attributed to the COVID-19 infection,^29^ which we have discussed. Symptoms of AC, PC, and LC may overlap with symptoms of menstrual pain, endometriosis, migraine, depression, anxiety disorder, and fibromyalgia and a correct allocation cannot always be made, even if a detailed medical evaluation was present. Furthermore, the patients with AC reported that they did not experience long-term symptoms after COVID-19 infection, but we cannot completely rule out that after completion of the survey, some symptoms reappeared/evolved, and they were therefore wrongly classified in the AC group but should have been in the LC group.

Another limitation is that our study does not include a control group of women without menstrual pain, which would have been interesting to ascertain significant differences between the groups. Future studies should be performed to fill this gap.

The strength of our study remains, including a relatively large sample of a group of subjects that have until now not been studied before. The study cohort is especially relevant since menstrual pain influences quality of life and female gender remains an important but understudied risk factor for developing LC that has still not been understood.

### Implications for practice and conclusion

Our study describes symptom awareness of endometriosis patients, which is crucial for patient characterization even beyond the pandemic. Patients with SCE or fibromyalgia in the cohort of patients with dysmenorrhea have an increased risk for developing LC. In patients with SCE the comorbidity of depression and anxiety disorder further increases the risk of experiencing LC. Future research should focus on underlying mechanisms linking severe dysmenorrhea with LC and if it is possible to reduce the risk of LC by improving the treatment of those pain and/or concomitant diseases.

## Data Access and Responsibility

The corresponding author had full access to all the data in the study and takes responsibility for the integrity of the data and the accuracy of the data analysis.

## Abbreviations Used

AC: Acute Covid-19, symptoms ≤ 4 weeks
ANOVA: Analysis of Variance
BMI: Body Mass Index
CI: Confidence interval
COVID-19: Corona Virus Disease 2019
DFG: Deutsche Forschungsgemeinschaft (German Research Foundation)
LC: Long Covid syndrome, symptoms ≥ 12 weeks
OR: Odds ratio
PC: Postacute Covid-19 syndrome, symptoms 4–12 weeks
SARS-CoV-2: Severe acute respiratory syndrome Coronavirus 2
SCE: Surgically confirmed endometriosis
SD: Standard deviation
SPSS: Statistics Statistical Package for Social Sciences
VAS: Visual Analogue Scale
